# Utility of sustainable ratio derivative spectrophotometry for the concurrent assay of synergistic repurposed drugs for COVID-19 infections; *Insilico* pharmacokinetics proof

**DOI:** 10.1186/s13065-024-01147-w

**Published:** 2024-03-07

**Authors:** Sara I. Aboras, Ahmed A. Megahed, Fawzy El-Yazbi, Hadir M. Maher

**Affiliations:** 1 Pharmaceutical Analytical Chemistry Department, Faculty of Pharmacy, University of Alexandria, Al-mesallah, Alexandria, 21521 Egypt; 2 Al-Basra Health Unit, Alamriya Medical Area, Ministry of Health, Alexandria, Egypt

**Keywords:** Fluvoxamine, Ivermectin, Repurposed, COVID-19, Synchronous, Spectrophotometry, Green, *Insilico*, Drug-drug interactions

## Abstract

The cutting-edge combination of fluvoxamine (FVM) and ivermectin (IVM) has been presented as a proposed dosage form for the treatment of COVID-19 infections in early diagnosed patients. The main objective of this work is to develop simple, sensitive, and efficient methods for the synchronous quantification of FVM and IVM without any prior separation. Four green UV-methods were employed for the synchronous quantification, namely: Fourier functions convolution of absorption spectra, FFAS, Fourier functions convolution of derivative spectra of absorption curves, FFDS, Fourier function convolution of ratio spectra of absorption curves, FFRS and the dual-wavelength method, DWM. FFRS and DWM approaches can be able to reconcile the two components' significantly interfering spectrum presented in this commixture. Good linearity was checked in the range of 5–40, and 2.5–25 μg/mL for the FVM, and IVM, respectively. All approaches developed have been recommended in compliance with ICH principles. Furthermore, the approaches’ greenness was predestined by “National Environmental Method Index” (NEMI), “Analytical GREEnness metric (AGREE)”, the “Analytical Eco-Scale”, and the “Green Analytical Procedure Index” (GAPI). In addition, spider diagram was utilized for the assessment of the greenness index of the solvent used. Beside greenness, the sustainability of our methods was investigated using the HEXAGON tool. Continuing the constant pursuit of greenness, drug-drug interactions (DDIs) between FVM & IVM were predicted by *insilico* tools to ensure the safety of the suggested mixture as a preliminary step before invitro and in vivo studies. Because they were deemed sustainable, affordable, and successful, the suggested UV-methods may be used for routine quality control investigations of the indicated formulations FVM & IVM.

**Supplementary Information:**

The online version contains supplementary material available at 10.1186/s13065-024-01147-w.

## Introduction

Coronavirus disease 2019 (COVID-19), caused by severe acute respiratory syndrome coronavirus 2 (SARS-CoV-2), was reported to be a pandemic disease all over the world. It constitutes a great burden on different global scales particularly the medical, social, and economical fields. SARS-CoV-2 is considered a rapidly changing virus which causes a wide range of pathological disorders, the most common of which are respiratory disorders and digestive tract disorders [1]. Patients with respiratory dysfunctions usually suffer from cough, fever, runny nose, which can proceed to shortness of breath and acute respiratory distress syndrome (ARDS) [2]. On the other hand, nausea, vomiting, and diarrhea are the main malfunctions associated with viral invasion of the alimentary canal [3]. Moreover, thyroiditis and possible autoimmune thyroid dysfunctions could be related to the viral infection [4]. Moreover, many post-COVID patients suffer from psychiatric disorders and impaired neuronal functions [5]. Although most patients with COVID-19 infections recover without marked complications, some cases are severely affected and need hospitalization in an attempt to control the virus-induced complications, e.g. ARDS, internal organ dysfunctions, with a probability of total organs’ failure and death [6].

It is noteworthy to mention that SARS-CoV-2 is an unprecedented virus with a wide range of diverse symptoms, treatment of such viral infection is gaining much interest in the medical field. Understanding the mechanism of action of SARS-CoV-2 virus in damaging body’s performance is very crucial in planning therapeutic protocols. It is reported that cytokine-related syndrome (CRS) is very common in infected patients [7]. This syndrome is accompanied with triggering inflammatory pathways followed by consequent induction of lung damage involving thromboembolism, pulmonary oedema, and lymphocytopenia [8, 9]. Viral-induced cytokine stroke can significantly cause severe damage of many internal organs [10, 11]. It has also been noticed that this virus can also result in fatal myocarditis in addition to different degrees of neuronal damage [5, 12].

In an attempt to combat such disastrous virus, vaccines have been introduced into the global market to decrease the probability/severity of viral invasion. However, production of vaccines is not an easy task, and it suffers from variability, difficulty in affordability, incompatibility issues, in addition to economic factors. In addition, emergence of mutant variables makes the efficiency of vaccines somewhat doubtful [13]. Accordingly, research focus on the use of therapeutic agents to combat such virus is a matter of public interest. Many therapeutic regimens were planned to target one or multiple sites in the viral life cycle and/or control viral-induced organs’ dysfunctions.

The cycle of developing therapeutic drugs is very tedious and time-consuming. It is well established that drug development and clinical evaluations are difficult processes that might take about ten to 12 years. Moreover, some methods of pharmacological therapy may take up to 15 years. Since antiviral therapeutic drugs with excellent specificity and efficacy against COVID-19 are urgently required, repurposing existing therapeutic drugs is a lucrative COVID-19 therapy method that can lessen the present strain on health care systems owing to the lengthy development delays of new drugs [14, 15].

**Fig. 1:**
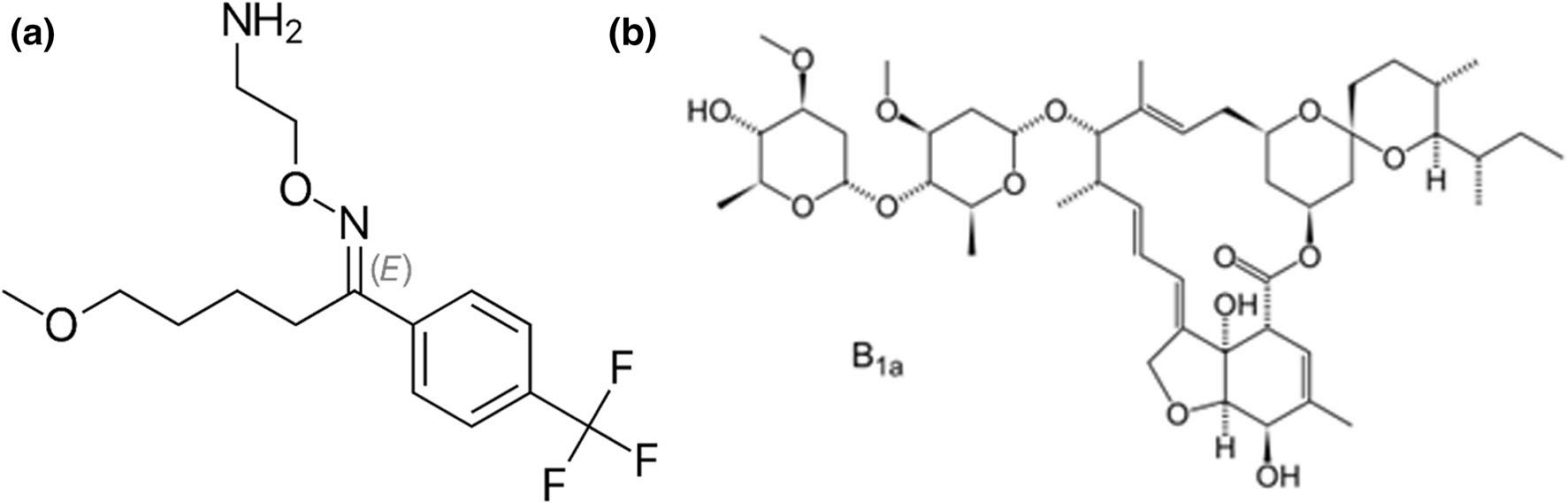
Chemical structures of (**A**) Fluvoxamine, and (**B**) Ivermectin

Antidepressants acting as selective serotonin reuptake inhibitors (SSRI) are among the repurposed medications in this field. The most common of which is fluvoxamine (FVM), a SSRI and a sigma 1 receptor (S1R) agonist, Fig. 1A [16]. Many studies were conducted to prove the anti-inflammatory and even the antiviral effect of FVM [17–20]. It was postulated that by inhibiting serotonin transport in the brain, an increased level of serotonin was recorded in the synaptic cleft with a marked anti-inflammatory effect and reduced platelet aggregation [17]. FVM diminishes the entrance of virus by inhibiting both acid sphingomyelinase enzyme as well as the formation of ceramide-enriched membrane domain. Being a sigma 1 receptor agonist, FVM attenuates viral replication with subsequent blocking of the formation of inflammatory cytokines, cytokine storm. In 2020, FVM has been clinically proved to be effective among high-risk outpatients with early diagnosed SARS-CoV-2 infection and it was effective in accelerating the recovery rate and decreasing the rate of viral progression and hospitalization [21–23].

Another outstanding example of repurposed drugs is ivermectin (IVM), Fig. 1B. Besides its outstanding antiparasitic effect, IVM has been investigated for other different clinical outcomes. In this respect, the antiviral effect of IVM against a wide range of viruses has been reported [24–26]. Recently, the efficiency of ivermectin against SARS-CoV-2 virus has been raised by researchers and public health providers [27]. The mechanism underlying the antiviral effect of IVM has been studied. It was postulated that ivermectin has a potential ionophoric effect which can induce ionic imbalance with further disruption in the integrity of the viral membrane [28, 29]. In addition, IVM has an anti-inflammatory effect which aids in diminishing the viral induced cytokine production and hence controlling severe complications associated with the virus [30]. It is also known to be immunomodulator with anticancer, glucose and cholesterol-lowering effect. Currently, IVM was found beneficial for low-risk outpatients with early diagnosed COVID-19 infection [31].

Combination therapies have been introduced to get a more effective viral inhibition especially with newly emerging mutant variants by affecting multiple viral targets, being of different modes of action, and also by counterfeiting complications induced by the viral infection. Combination therapies have the advantages of being more therapeutically effective compared with its individual components with decreased doses and less side effects [32, 33]. In this respect, a combination of FVM and IVM has been recommended for the treatment of early-diagnosed patients [34]. This combination has recently been reported to have synergistic effect in fighting SARS-CoV-2 virus with acceptable tolerability and minimal side effects as a result of reduced doses. The two drugs act synergistically to encompass the invading virus with a strong relation of decreasing hospitalization with the prevention of “long haul COVID” [34].

Polypharmacy is the concept involving simultaneous use of several drugs which could result in undesirable drug-drug Interactions (DDIs) in the human body. There are three types of DDIs: pharmaceutical, pharmacodynamic, and pharmacokinetic. Pharmaceutical DDIs occur when drugs are mixed inappropriately in liquid forms before administration, while pharmacodynamic DDIs occur when co-administrated drugs act on the same target. The current study focuses on pharmacokinetic (PK) DDIs linked to alterations in drug metabolism conducted by Drug-Metabolizing Enzymes (DMEs). Many DDIs are induced by changes in one drug's plasma concentrations because of another drug blocking and/or stimulating the object drug's metabolism or transporter-mediated disposition. The number of conceivable combinations of several thousand approved and repurposed pharmaceuticals is tremendous, as is the number of potentially harmful drug interactions. Therefore, conducting scientific testing of the safety and efficacy of all medication combinations is unfeasible. As a result, computational methods for DDIs prediction must be applied to prioritize the list of probable DDIs. This will be followed by selecting specific in vitro assays, then in vivo research, and optimizing the design of experimental testing and clinical trials [35–37].

Literature review reveals that different methods have been reported for the determination of either IVM or FVM alone or in combination with other drugs in their pharmaceutical dosage forms. IVM was determined in its tablets using HPLC [38, 39], HPTLC [40], and spectrophotometric methods which include direct absorbance [37] and multivariate calibration [41]. Also, determination of FVM in its pharmaceuticals was reported using HPLC [42], TLC [43], in addition to electrochemical methods [44, 45]. Fluorimetric determination of FVM was also reported following its derivatization with fluorogenic reagents [46, 47]. Spectrophotometric determination of FVM was based on its reaction with different chromogenic reagents with the formation of colored products [48–50].

To our knowledge, literature review reveals the absence of any reports for the simultaneous determination of IVM and FVM. The great difference in their therapeutic doses, being taken in the ratio of 100: 12 for FVM and IVM, respectively, makes their simultaneous determination a tackling task. Therefore, this work will focus on the development and validation of spectrophotometric methods for the simultaneous determination of the cited drugs without prior separation. Compared with other analytical techniques, spectrophotometry has many advantages regarding simplicity, cost effectiveness, less time consumption, ease of application, less practical work, besides the availability of instruments. Spectrophotometric determination of multi-component mixtures with no prior separation usually requires some sort of spectral mathematical treatment. In spite that derivative spectrophotometry can remove many types of errors, sometimes it could not resolve high degree of spectral overlap [51]. Derivative treatment of ratio spectra of absorption curves, referred to as derivative ratio spectra (DRS) has also been applied to resolve complex mixtures [51–53]. Moreover, spectral convolution with combined trigonometric Fourier functions has been widely used in the determination of pharmaceutical mixtures. This includes convolution of zero-order absorption spectra [52], referred to as Fourier functions absorption spectra (FFAS), or of ratio spectra of the absorption curves, the so-called Fourier functions ratio spectra (FFRS) [51, 52]. Also, Fourier functions convolution has been extended to absorbance ratio spectra obtained using a double divisor for the determination of ternary mixtures which was referred to as the hybrid double divisor-ratio spectra method (HDDR) [53]. Application of Fourier functions convolution to different types of spectral data is highly beneficial in eliminating different types of interferences and resolving spectral overlap [51–53]. In addition, the dual wavelength method (DWM), has gained interest in resolving spectral interference without any complicated mathematical treatment of data [54]. This method is based on selecting two spectral data points which difference is dependent only on a particular component with no interference of the other [54].

This work aims at development and validation of simple, cost-effective, and earth-friendly spectrophotometric methods for the simultaneous determination of a novel anti-COV-2 binary mixture comprising of ivermectin and fluvoxamine. These methods included Fourier functions convolution of absorption spectra, FFAS, Fourier functions convolution of derivative spectra of absorption curves, FFDS, and Fourier function convolution of ratio spectra of absorption curves, FFRS. In addition, the dual-wavelength method, DWM, was also used. The proposed methods were successfully applied to the determination of mixtures of the cited drugs in laboratory-prepared mixtures.

Moreover, the degree of methods’ greenness was validated using different assessment tools namely, “National Environmental Method Index” (NEMI), “Analytical GREEnness metric (AGREE)”, and the “Analytical Eco-Scale”, the “Green Analytical Procedure Index” (GAPI) [55–58]. As well, the methods’ sustainability was assessed using the Hexagon tool [59]. The selection of the environmental solvent is crucial for obtaining optimal greenness analytical methodologies, therefore the impact on safety, health, and the environment (SHE) must all be considered when evaluating the solvent’s greenness. Tool like the Spider diagram for the assessment of the greenness index has helped achieve this [60–63].

In addition, this work includes evaluation of possible DDIs between FVM and IVM using *insilico* techniques which were applied using the PASS (Prediction of Activity Spectra for Substances) program and PoSMNA (Pairs of Substances Multilevel Neighborhoods of Atoms) substructural descriptors. These models were able to predict the severity classes of DDIs for pairs of molecules using OpeRational ClassificAtion (ORCA). ORCA divides DDIs into five classes: contraindicated (class 1), provisionally contraindicated (class 2), conditional (class 3), minimal risk (class 4), no interaction (class 5). Besides, the seven most important P450 cytochromes are involved in this computer model for DDI prediction: CYP1A2, CYP2B6, CYP2C19, CYP2C8, CYP2C9, CYP2D6, and CYP3A4.

## Experimental

### Apparatus

A UV–Vis double beam spectrophotometer (Shimadzu UV-1800 PC, Kyoto, Japan) equipped with quartz cells with matching 1 cm path lengths was used to conduct the spectrophotometric measurements. The UV probe software was employed for data analysis.

The models used for *insilico* DDIs are freely available through the Internet on the Way2Drug.com web portal on the DDIs web-service [64], which allows for the prediction of various DDIs parameters with no pre-registration.

### Materials and reagents

IVM and FVM reference standards were supplied from Sigma Aldrich with certified purity of 99.2% and 99.6%, respectively. Methanol (Baker, Ireland) was utilized in the study.

### Preparation of stock and standard solutions

Stock solutions of FVM or IVM were prepared by dissolving 25 mg of each drug in separate 25 mL volumetric flask with methanol to obtain stock solutions with a concentration of 1 mg/mL of each drug. These stock solutions were kept refrigerated for 7 days. Stock solutions were then diluted with methanol to obtain a series of working standard solutions within the concentration range of (5–40 µg/mL) and (2.5–25 µg/mL) for FVM and IVM, respectively.

### Preparation of laboratory prepared mixtures

Accurate volumes of FVM and IVM stock solutions were accurately transferred into a set of 10-mL volumetric flasks. Final volumes were made with methanol to get mixtures with final concentrations of (5–40 µg/mL) and (2.5–25 µg/mL) for FVM and IVM, respectively. Three mixtures of different concentrations of the analyzed drugs were prepared as follows: 20 and 2.5 µg/mL, mix (1), 10 and 10 µg/mL, mix (2), and finally 5 and 15 µg/mL, mix (3), for FVM and IVM, respectively.

### Construction of calibration curves

#### Fourier functions convolution of absorption spectra, FFAS

Zero-order absorption spectra of working standards of both drugs were convoluted using combined trigonometric Fourier functions. Fourier function coefficients for eight equally spaced wavelengths measured at 1 nm interval, t’_r_, were calculated using the following equation:

1 $$t^{\prime } _{r} = \,[( + 1.707)Ar_{0} + ( + 0.707)Ar_{1} + ( - 0.707)Ar_{2} + ( - 1.707)Ar_{3} + ( - 1.707)Ar_{4} + ( - 0.707)Ar_{5} + ( + 0.707)Ar_{6} + ( + 1.707)Ar_{7} ]/4$$

where Ar_0_-Ar_7_ represent eight zero-order absorption values. The numbers between brackets refer to the specified combined Fourier functions calculated as follows [50, 51, 53]:

2 $$T^{\prime } = [cosx + cos(x + 45)]$$

Fourier function coefficients were recorded at 270 nm and was used for the determination of FVM. Calibration curves and the regression equations derived therefrom were constructed by relating the Fourier functions coefficients calculated at the selected point to the corresponding FVM concentration.

#### Fourier functions convolution of derivative spectra of absorption curves, FFDS

Zero-order absorption data were used to derive the second derivative spectra, at 3 nm interval, which were then convoluted using the trigonometric Fourier functions, exactly as mentioned above. Fourier functions coefficients were recorded at 240 nm, for IVM determination.

#### Fourier functions ratio spectra, FFRS

Ratio spectra were first calculated by dividing the absorption spectra of the standard solutions of IVM and FVM by that of the standard spectrum of 10 µg/mL IVM or FVM for the determination of FVM and IVM, respectively. The derived absorbance ratio spectra were then convoluted with the trigonometric Fourier functions, exactly as mentioned above. Fourier functions coefficient were measured at the selected points, 272 nm and 258 nm (peak to peak) for FVM determination, 254 nm and 291 nm (peak to peak) for IVM determination. By analogy, calibration graphs and the corresponding regression equations were derived by relating the Fourier functions coefficients calculated at the selected points to the corresponding concentration.

#### Dual wavelength zero-order method, DWZ

Zero-order absorption spectra of the standard solutions were scanned in the 200–400 nm range. Absorbance values were measured at 267 and 290 nm for FVM determination and at 231 and 268 nm for IVM determination. FVM was calculated by plotting the calibration curve between the difference in the absorbance values at 267 and 290 nm (zero difference for IVM) against the corresponding concentration. However, IVM was derived by calculating the difference in absorbance values between 231 and 268 nm (zero for FVM) against the corresponding concentration.

### Analysis of laboratory-prepared mixtures

The absorbance spectra of the prepared mixtures were measured spectrophotometrically and then handled in as previously mentioned. The concentrations of each drug in the analyzed mixtures were calculated from the corresponding regression equations.

## Results and discussion

The combination of FVM and IVM therapy demonstrated a synergistic effect in the battle against the SARS-CoV-2 virus [34]. Simultaneous determination of the two drugs in combined mixtures is extremely important for quality control purpose. Spectrophotometry was proposed as a cheap, easily applicable with low time consumption analytical method.

### Spectrophotometric characteristics

**Fig. 2:**
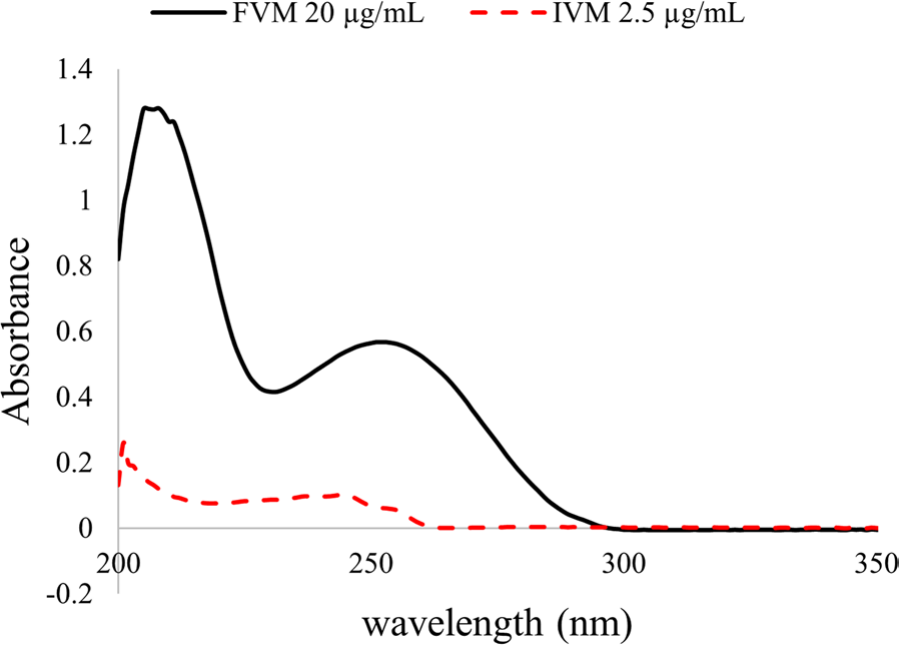
Zero-order spectra of FVM (20 μg/mL) and IVM (2.5 μg/mL) using methanol as a blank

The zero-order spectra of FVM and IVM showed a significant overlap, Fig. 2, particularly that there is a great difference in their therapeutic doses, being taken in the ratio of 100: 12 for FVM and IVM, respectively, which adds to the difficulty in their simultaneous determination. As a result, simultaneous estimation of this binary combination was challenging. Accordingly, different types of mathematical treatment of absorbance data were tried for the purpose of spectral resolution with no need of pre-separation procedures. Four methods were proposed, three of which depend on spectral convolution with trigonometric Fourier functions of absorption spectra, FFAS, derivative spectra of absorption curves, FFDS, and ratio spectra of absorption curves, FFRS. In addition, the dual-wavelength method, DWM, was also proposed.

In this respect, it is noteworthy to mention that preliminary trials were based on investigating the derivative zero crossing technique. Despite being the simplest among chemometric techniques, unfortunately, neither first nor second derivatives could determine IVM in combinations of FVM due to lack of zero-crossing points of the latter, Additional file 1: Figs. S1, S2. Although first and second derivative values at 270 and 284, respectively, could be used for selective determination of FVM, the signal to noise ratio induced by derivative calculations resulted in a decrease in the sensitivity of these methods, Additional file 1: Fig S1, S2. Moreover, ratio spectra obtained from zero-order absorption spectra of the selected drugs, as mentioned later, were used to derive the corresponding first and second derivative spectra, the so-called ratio derivative method. In this method, both FVM and IVM could be solved but with lower sensitivity compared to the proposed FFRS, Additional file 1: Figs S3, S4. As a result, Fourier functions were used to resolve spectral overlap with acceptable determination of FVM and IVM in their binary mixtures with higher sensitivity.

### Methods optimization

#### Fourier functions of absorption spectra (FFAS)

**Fig. 3:**
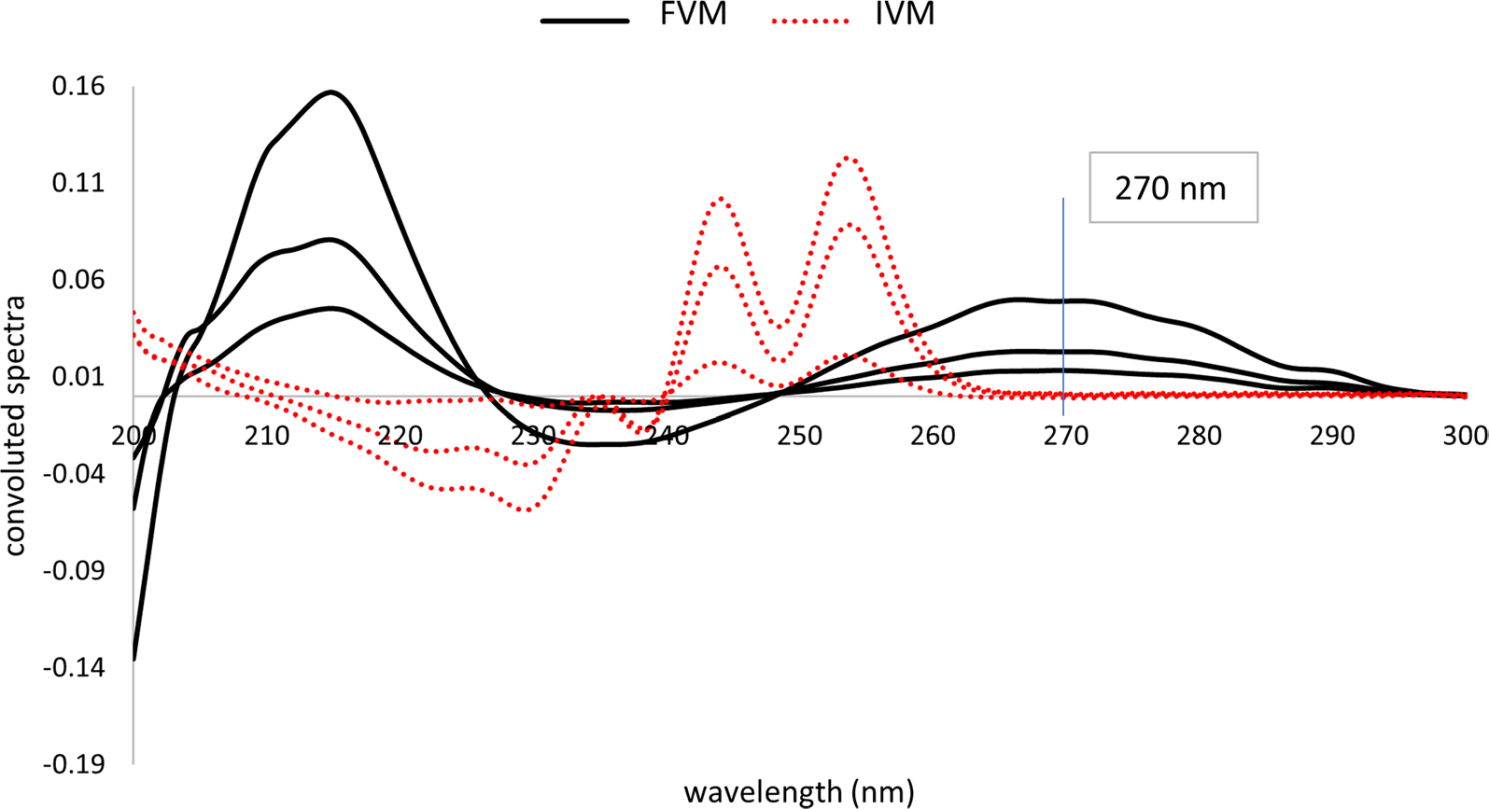
Fourier convoluted zero-order spectra of different concentrations of FVM (5, 10, 20 μg/mL) and IVM (2.5, 10, 15 μg/mL) showing zero crossing of IVM at 270 nm

To begin with, zero-order absorption spectra of both FVM and IVM were convoluted using eight points trigonometric Fourier functions with an interval of 1 nm. Convoluted spectra were obtained by plotting the calculated coefficients as a function of wavelength, taken as the mean value, Fig. 3. As seen from this figure, FVM could be determined at 270 nm where the contribution of IVM at this point was zero. However, no points showed zero contribution of FVM, thus this method could not be used for the determination of IVM.

#### Fourier functions of derivative spectra (FFDS)

**Fig. 4:**
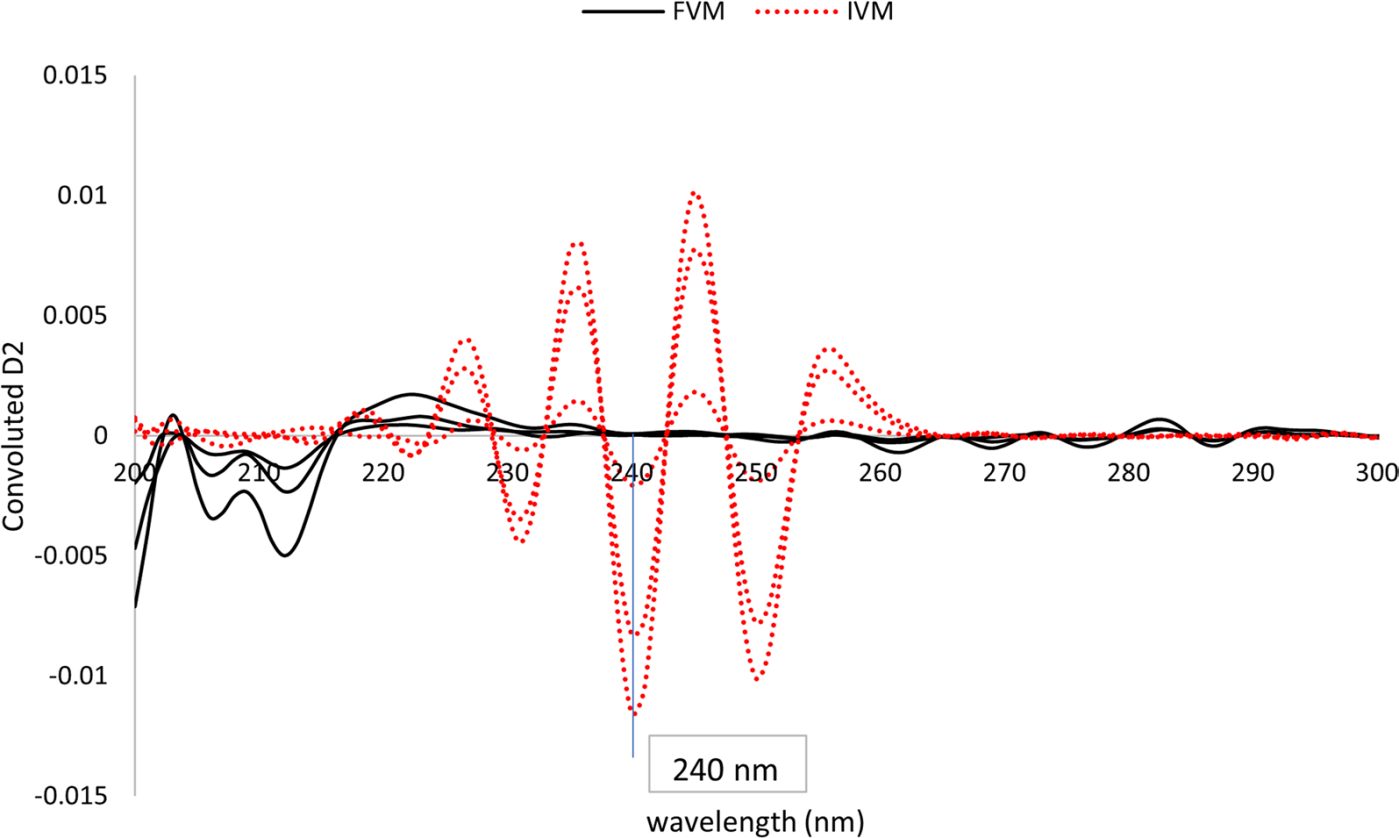
Fourier convoluted second-derivative spectra of different concentrations of FVM (5, 10, 20 μg/mL) and IVM (2.5, 10, 15 μg/mL) showing zero crossing of FVM at 240 nm

In this method, zero-order absorption spectra of both analytes were initially used to derive first and second derivative spectra which were then convoluted exactly as in FFAS. Convoluted first derivative spectra did not show any points of a signal of one compound while zero contribution of the other. On the other hand, convoluted second derivative spectra enabled the determination of IVM by measuring the derivative values at two points 240 and 245 nm, peak to peak, which showed zero contribution of FVM at both points, Fig. 4. It is also clear that FVM could not be determined using this method since no points could be seen in the spectra where a signal of FVM corresponded to nil contribution of IVM.

It is noteworthy to mention that second derivative spectra were derived using ∆λ of 3 nm followed by Fourier function convolution with ∆λ of 1 nm since the wavelength interval is a very important optimization parameter that could significantly affect the noise level and hence the sensitivity of the method.

#### Fourier functions of ratio spectra of absorption curves spectra (FFRS)

**Fig. 5:**
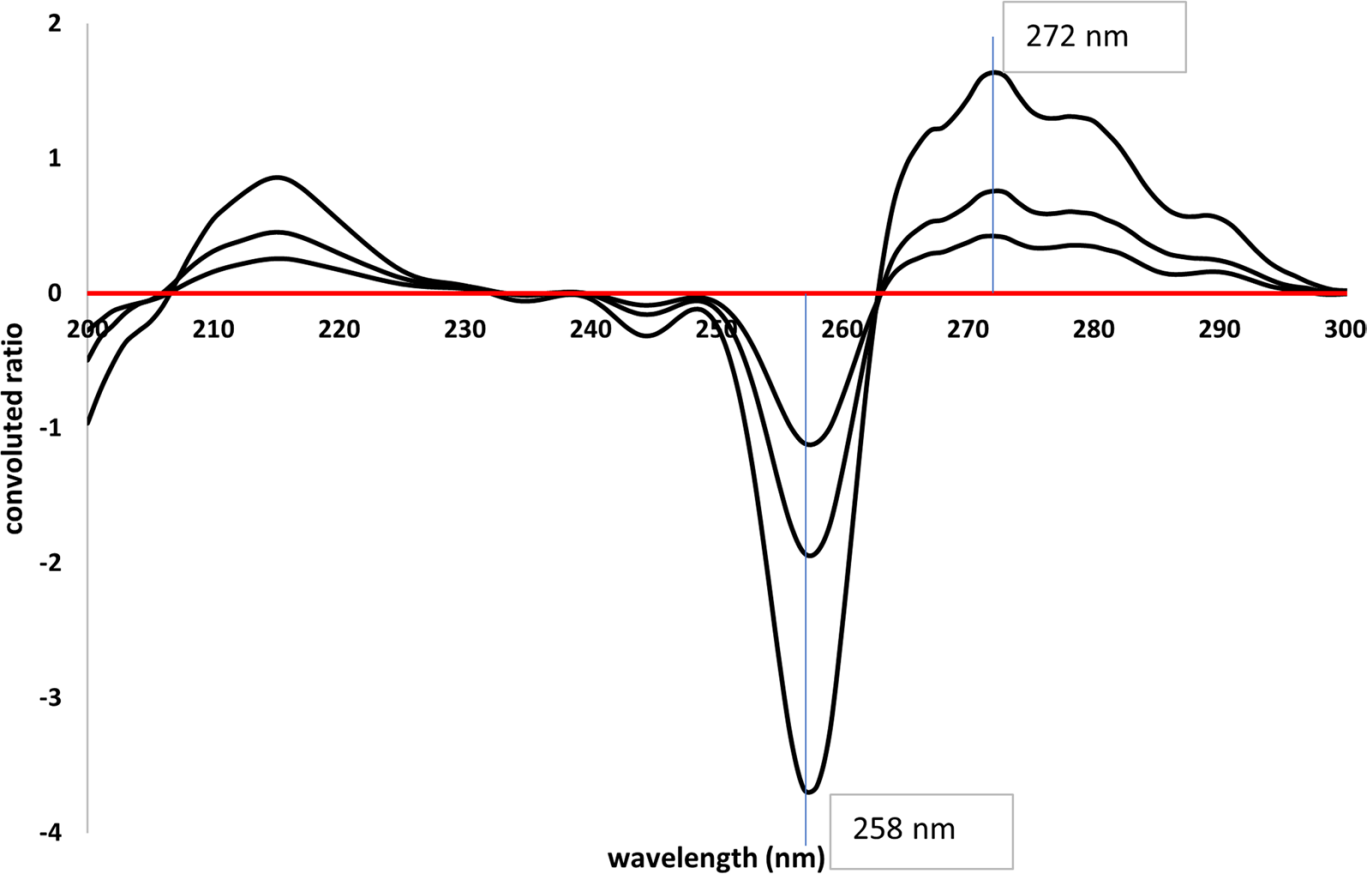
Fourier convoluted ratio spectra (I) of different concentrations of FVM using 10 μg/mL IVM as divisor

**Fig. 6:**
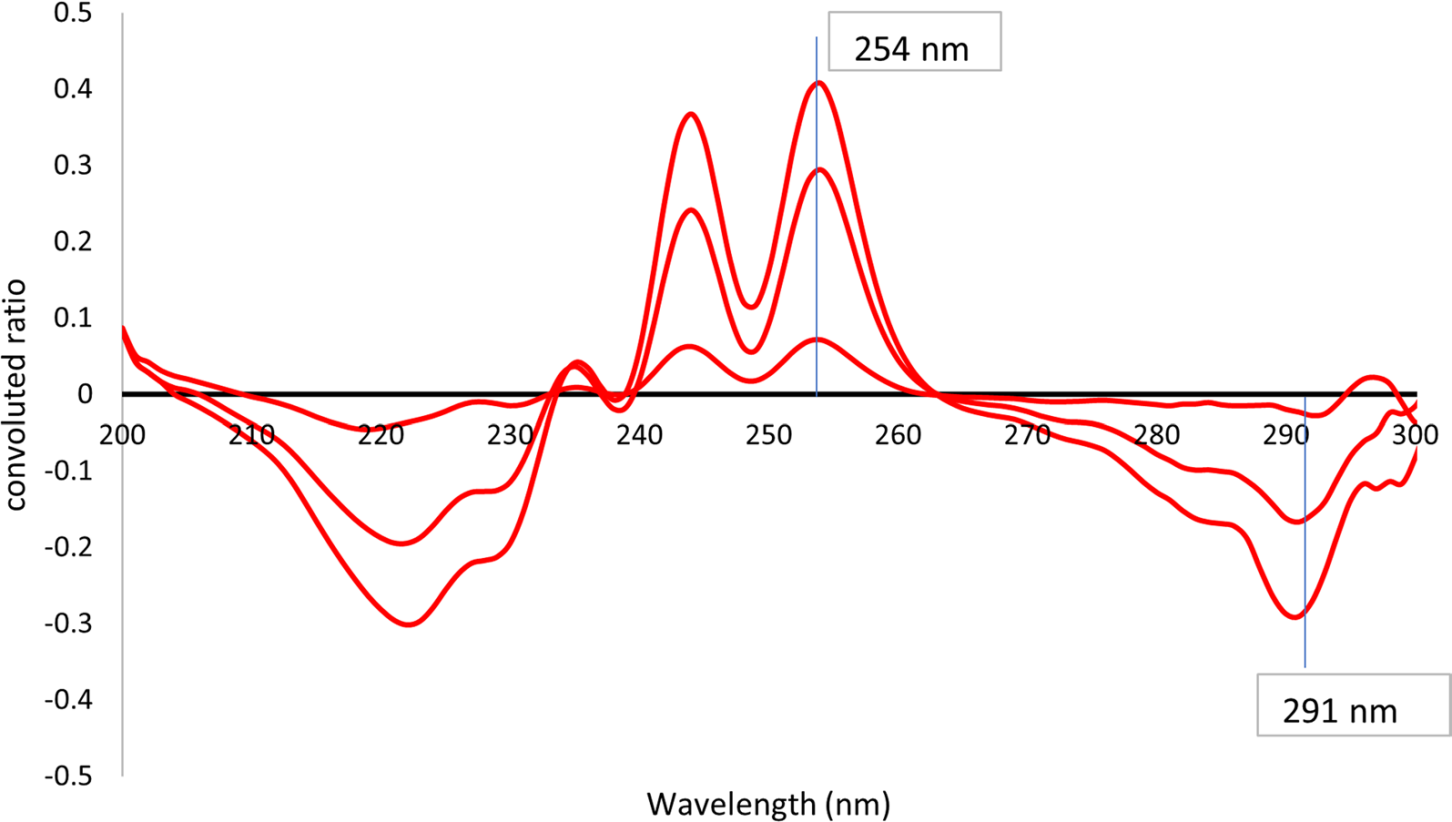
Fourier convoluted ratio spectra (II) of different concentrations of FVM (5, 10, 20 μg/mL) using 10 μg/mL FVM as divisor

**Fig. 7:**
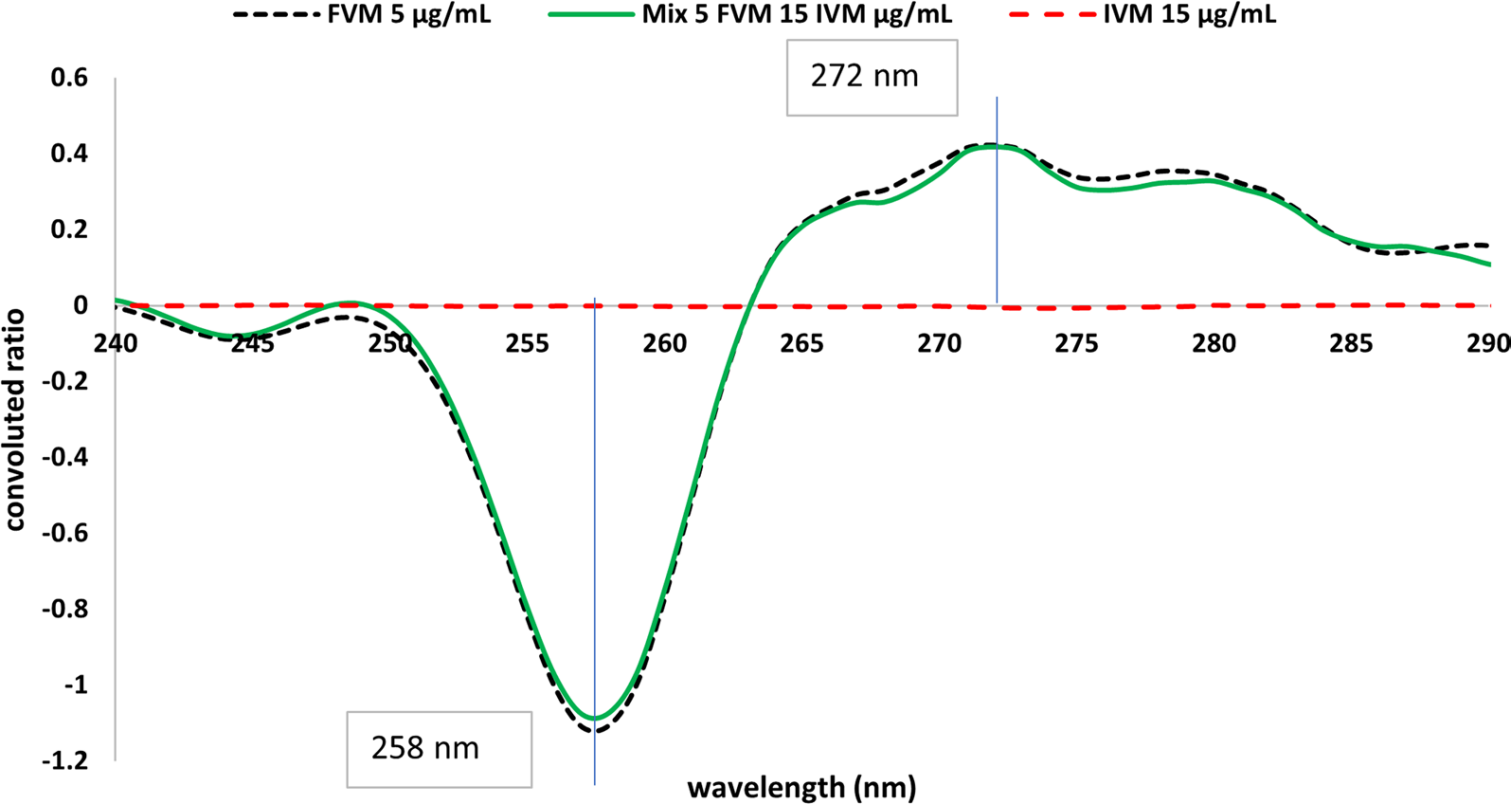
The coincident convoluted ratio spectra of FVM 5 μg/mL, IVM 15 μg/mL and Mix of both using 10 μg/mL IVM as divisor

**Fig. 8:**
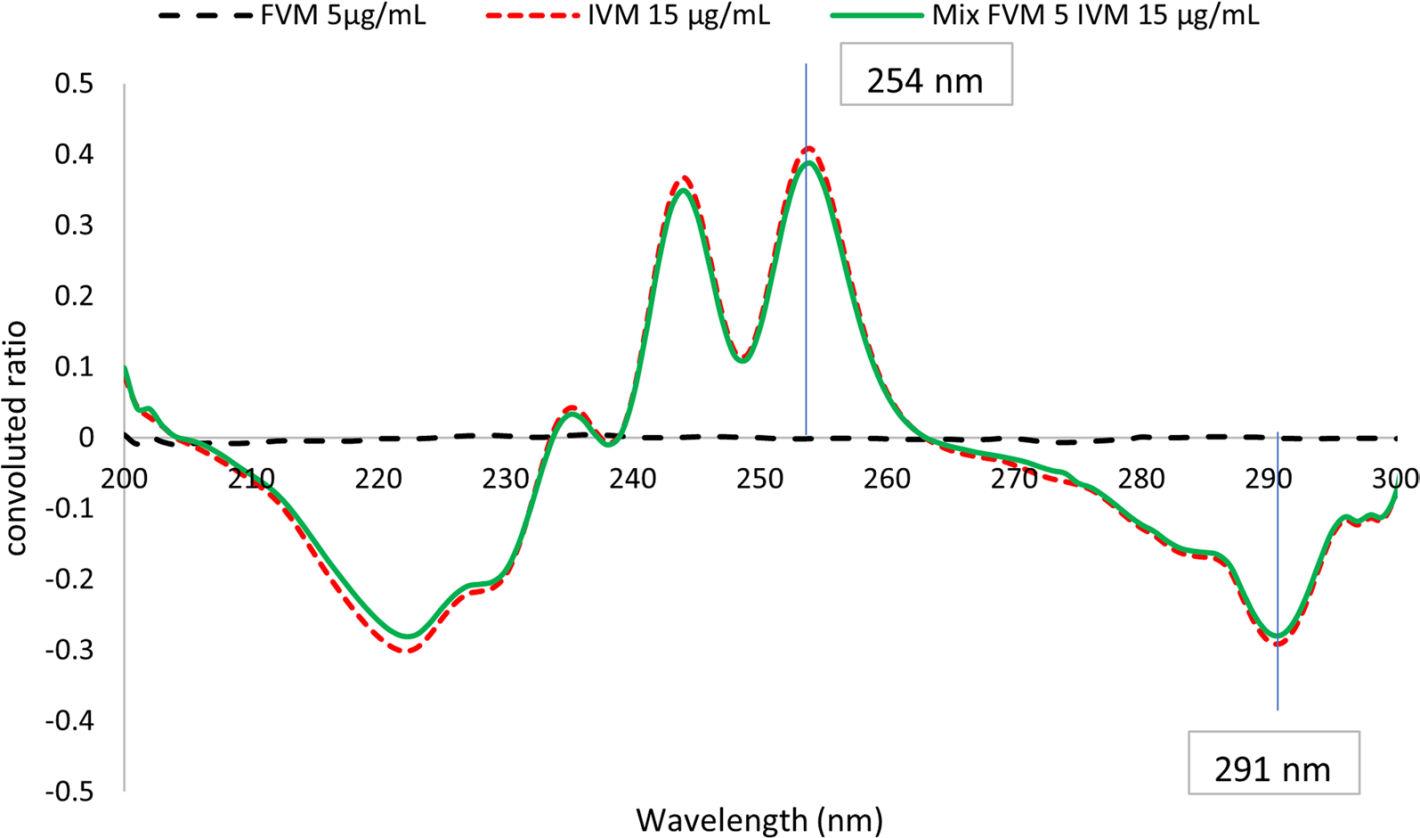
The coincident convoluted ratio spectra of FVM 5 μg/mL, IVM 15 μg/mL and Mix of both using 10 μg/mL FVM as divisor

This method was based on two consequent mathematical treatments of absorption spectra of both analytes. At first, ratio spectra were derived by dividing absorption spectra by the divisor, 10 μg/mL FVM, for the selective determination of IVM, or by the divisor, 10 μg/mL IVM, for the determination of FVM. Secondly, the obtained ratio spectra were convoluted using eight points trigonometric Fourier functions at 1 nm interval. Since the divisor concentration is the key parameter affecting the shape of the ratio spectrum, various concentrations of FVM (5, 10, and 20 μg/mL) and IVM (2.5, 10, and 15 μg/mL) were tested. Divisors’ concentrations of (10 μg/mL) FVM and (10 μg/mL) IVM were used since experimental trials revealed that they produced the least level of noise with the best sensitivity. The corresponding FFRS convoluted curves were obtained by plotting the calculated Fourier function coefficients against wavelength, taken as mean values, Fig. 5. It is clear from Fig. 5 that the coefficients selected at 272.0 and 258.0 nm (peak to peak) could be used for measuring the concentration of FVM, while coefficients at λ 254.0 and 291.0 nm (peak to peak) could be equivalent to IVM concentration, Fig. 6. In this method, selection of the working wavelengths was based on points of coincidence between the spectra of the standard solution of one compound and the binary mixture containing the same concentration of the specified compound, Figs. 7 and 8. This enables the use of peak-to-peak measurements and hence increases the sensitivity of the analytical signals.

As mentioned before [50–53], FFAS could be successfully used as a more sensitive alternative to derivative spectrophotometry. The potential of Fourier functions convolution in eliminating different types of interferences makes it highly beneficial in solving binary mixtures with significantly overlapping spectra, even at minor concentrations. Optimum selection of the type of function, number of data points, and wavelength interval guarantees high level of selectivity for a particular compound with no interference of the other. In the present study, FFAS, FFDS, FFRS, cosine function using eight points was found optimum, $T{\prime}\hspace{0.17em}=\hspace{0.17em}[cos x\hspace{0.17em}+\hspace{0.17em}cos (x\hspace{0.17em}+\hspace{0.17em}45)]$. In addition, a wavelength interval of 1 nm was found ideal for the analysis.

#### Dual wavelength zero-order method (DWZ)

**Fig. 9:**
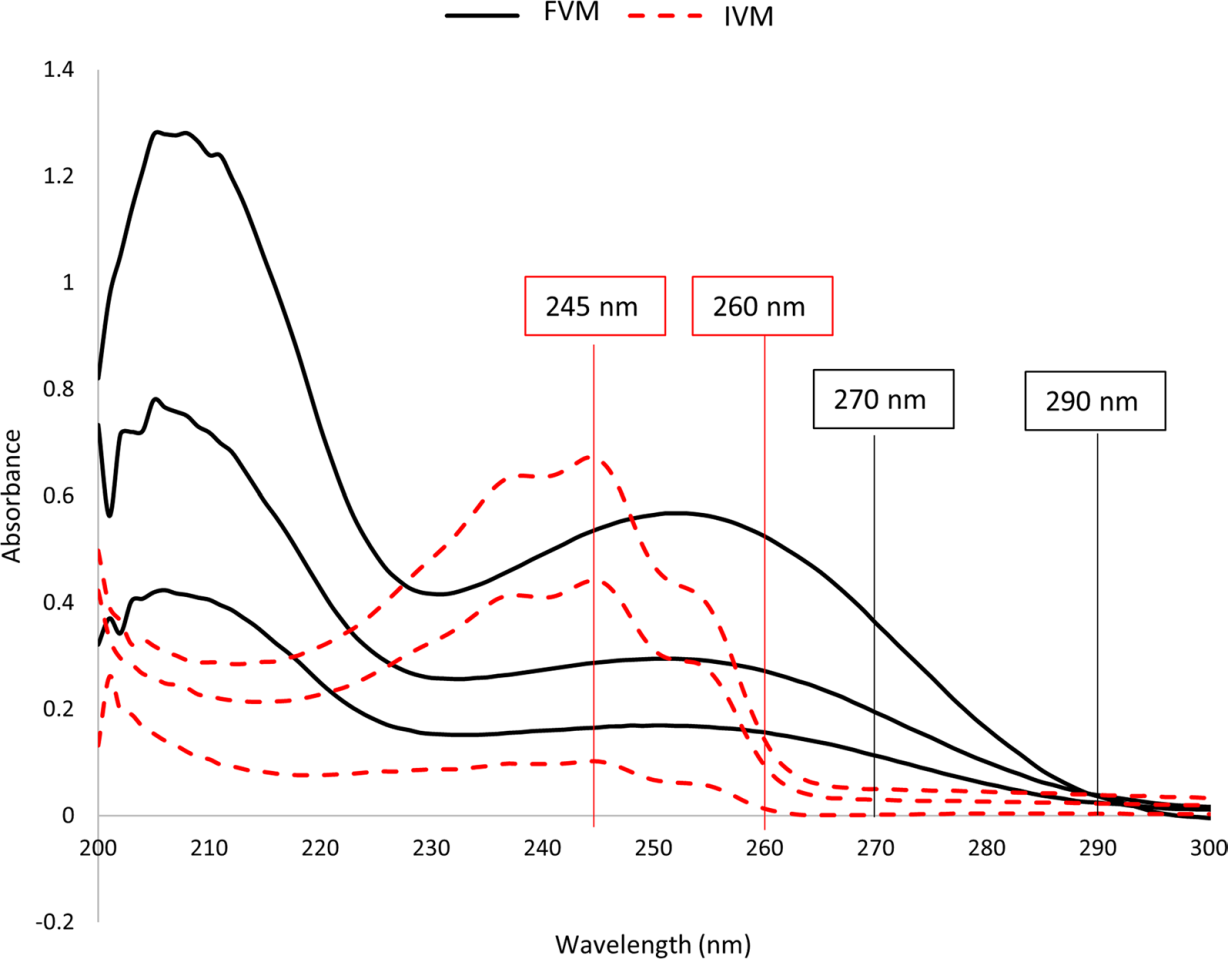
Zero-order spectra of FVM (5, 10, 20 μg/mL) and IVM (2.5, 10, 15 μg/mL) showing the selected wavelengths for DWZM using methanol as a blank

This method relies on choosing two wavelengths on the absorption spectra which shows a markable difference for one component while zero difference for the other. In this case, the absorbance difference of the compound of interest is proportional to its concentration, with absence of interference from the other compound. As shown from the absorption spectra of FVM and IVM, Fig. 9, the difference in absorbance values at the selected wavelengths of 267 and 290 nm can be used for the determination of FVM, with zero contribution of IVM, while those at the wavelengths of 231 and 268 nm were selected for the determination of IVM, with no interference from FVM. DWM is also known for its potential to cancel any constant interference by subtraction [54]. Calibration graphs for FVM and IVM were derived by relating the absorbance differences at the selected wavelengths against the corresponding concentration.

### Methods validation

Validation of the proposed methods was evaluated in accordance with ICH guidelines [65] as follows:

#### Linearity

The calculated Fourier function coefficients for FFAS, FFDS, and FFRS, or absorbance difference for DWM method, each selected at the prespecified points, were related to the concentrations of FVM and IVM. In each case, high degree of linearity was assessed by calculating regression coefficient values which were higher than 0.999, for all the proposed methods (Table 1). Other statistical parameters were also calculated including standard deviations of residuals (_Sy/x_), of intercept (S_a_), and of slope (S_b_).

**Table 1:** Analytical performance data of the calibration graphs for the determination of FVM, and IVM by the proposed methods

Analyte	FVM			IVM		
Method	FFZD	FFR	DWZ	FFZD	FFR	DWZ
Wavelength (nm)	270	272–258	∆A 270–290	240	254–291	∆A 245–260
Linearity range (µg/mL)	5–40			2.5–25		
LOD (µg/mL)^a^	0.7352	1.5306	0.4202	0.3995	0.0980	0.1213
LOQ (µg/mL)^b^	2.2279	4.6381	1.2732	1.2106	0.2971	0.3677
Regression coefficient	0.99929	0.99966	0.99963	0.99947	0.99997	0.99995
Slope	0.0025	0.2521	0.0145	0.0008	0.0483	0.0350
SE of slope	0.0001	0.0114	0.0002	0.00001	0.0002	0.0002
Intercept	− 0.0011	0.2284	0.0130	0.0001	-0.0249	0.0005
SE of intercept	0.0006	0.1169	0.0019	0.0001	0.0014	0.0013

^a^Limit of detection

^b^Limit of quantitation

#### Limit of quantitation (LOQ) and limit of detection (LOD)

The following equations were used to determine LOD and LOQ in accordance with ICH recommendations [55]:

LOD = 3.3 SD/Slope, LOQ = 10 SD/Slope, where SD represents the intercept’s standard deviation.

The great sensitivity of the derived methods was supported by the low values of LOD and LOQ (Table 1). The most sensitive method is DWZ with LOD and LOQ 0.4202 and 1.2732 μg/mL and 0.1213 and 0.3677 μg/mL for FVM and IVM, respectively.

#### Accuracy

Accuracy of the proposed methods were estimated by analyzing mixtures of FVM and IVM prepared within the linearity range of each compound, with different ratios as mentioned. Good mean percentage recoveries of the suggested methods (Table 2) imply excellent accuracy.

**Table 2:** Assay results for the determination of FVM, and IVM in pure forms

% Recovery^a^						
Analyte	FVM^b^			IVM^c^		
Method	FFZD	FFR	DWZ	FFZD	FFR	DWZ
	100.42	100.79	99.89	100.42	100.79	99.89
	99.68	100.28	99.98	98.73	98.83	98.16
	99.47	100.62	100.20	99.47	100.62	100.20
Mean ±	99.86 ±	100.56 ±	100.02 ±	99.54 ±	100.08 ±	99.41 ±
SD	0.50	0.26	0.16	0.85	1.09	1.10

^a^The average of the three determinations

^b^Amount taken of 10, 20, and 30 μg/mL

^c^Amount taken of 5, 10, and 20 μg/mL

#### Precision

To examine the intraday and interday precision, solutions of three different concentrations of FVM (10, 20, and 30 μg/mL) and IVM (5, 10, and 20 μg/mL) were analyzed in triplicates on the same day and on three successive days, respectively. Found concentrations derived from the respective regression equations were used to calculate % RSD which did not exceed 2% showing high degree of precision of the developed methods.

#### Selectivity

To assess the selectivity of the proposed methods within the linearity limits, various laboratory-prepared combinations containing various ratios of FVM and IVM were examined: 20:2.5, 10:10, and 5:15 μg/mL, respectively. Table 3 demonstrated excellent mean percentage recoveries for FVM and IVM with low standard deviation values using the four proposed spectrophotometric methods, FFAS, FFDS, FFRS, and DWZ.

**Table 3:** Assay results for the determination of FVM, and IVM in synthetic mixtures

% Recovery^a^						
Analyte	FVM			IVM		
Method	FFZD	FFR	DWZ	FFZD	FFR	DWZ
Mix 1^b^	100.42	100.79	99.89	100.42	100.79	99.89
Mix 2^c^	99.68	100.28	99.98	98.73	98.83	98.16
Mix 3^d^	99.47	100.62	100.20	99.47	100.62	100.20
Mean ±	99.86 ±	100.56 ±	100.02 ±	99.54 ±	100.08 ±	99.41 ±
SD	0.50	0.26	0.16	0.85	1.09	1.10

^a^The average of the three determinations

^b^Mix 1 containing 20 FVM and 2.5 IVM μg/mL

^c^Mix 2 containing 10 FVM and 10 IVM μg/mL

^d^Mix 3 containing 5 FVM and 15 IVM μg/mL

#### Statistical comparison between the developed methods

In order to verify the results of the proposed methods, one-way ANOVA [66] test was computed to three sets of three samples for both drugs in the three synthetic mixtures. Therefore, Snedecor’s F-values were determined and compared with the standard tabulated value (p = 0.05). The calculated F values of 3.55 and 0.36 for FVM and IVM assay, respectively, did not exceed the critical value (5.14), indicating that absence of major differences between the proposed methods at p = 0.05 (Additional file 1: Table S1).

### Assessment of greenness and sustainability of the proposed analytical methods

#### Greenness assessment

**Fig.10:**
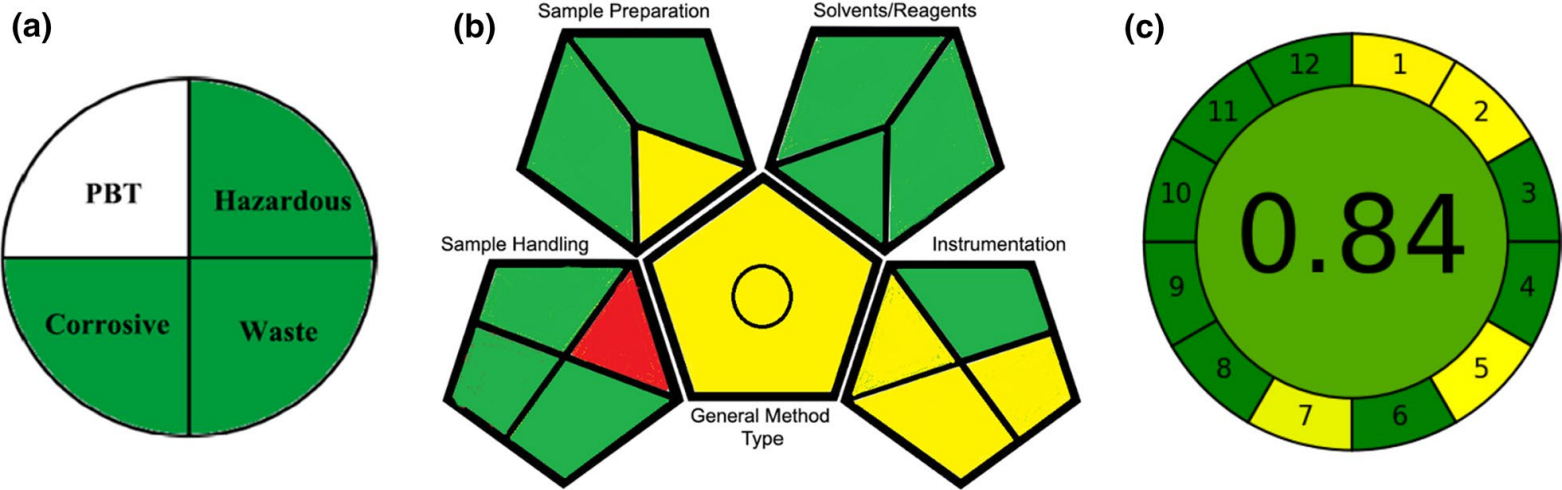
The greenness assessment tools of the proposed spectrophotometric methods using NEMI, GAPI, and AGREE

Greenness assessment was performed using the National Environmental Methods Index (NEMI) [55], a circular pictorial representation divided into four quadrants. In this method, the list of poisonous, bio-accumulative, and persistent substances (PBT) is depicted in the top quadrant while the second quadrant represents the use of hazardous wastes as per the Environmental Protection Act. However, the third quadrant refers to the use of corrosive agents having a pH range of 2 to 12. In addition, waste production is evaluated in the fourth quadrant, less or more than 50 mL or grams. Since the proposed spectrophotometric methods used methanol as a solvent, the first quadrant was highlighted in white, being PBT, and the second quadrant was highlighted in green, not among the EPA’s list of hazardous compounds. Given that methanol had a pH range of 2 to 12, the third quadrant was colored green. And since the residual volume of solvents resulted from the analysis of one analytical sample was less than 50 mL, the final quadrant was colored green. Figure 10a shows the overall NEMI pictogram for the suggested techniques.

Moreover, Gauszka et al. proposed the Analytical Eco scale (ESA) tool in 2012 [56] for assessing the degree of greenness of analytical procedures, and it is based on the production of a numerical total score that classifies the level of greenness of the examined analytical procedure. A perfect green process receives a score of 100 and incurs no penalties. Our methods’ ESA score was 89 with 11 penalties which indicates the sustainability of the mathematical methods, Table 4.

**Table 4:** The penalty points of the proposed spectrophotometric method according to the Analytical Eco-scale

Reagents/instruments	Penalty points (PPs)
Methanol	6
Energy of spectrophotometer	0
Occupational hazard	0
waste	5
PPs	**11**
Eco-scale score	**89**

The green analytical process index (GAPI) [57] is a qualitative statistic for assessing the greenness of analytical procedures. The GAPI value is color coded in the final pictogram. It is believed that GAPI is more advantageous compared with the NEMI pictogram since it has 15 evaluation stages and three times as many components. It also incorporates the revised NEMI principles, making it outperform NEMI in the evaluation of green analytical chemistry. The final GAPI pictogram was displayed in Fig. 10b with 9 green, 5 yellow and 1 red zone, indicating high greenness of the proposed methods.

In June 2020, AGREE [58], a free greenness assessment tool, was introduced based on the twelve GAC fundamentals. The total score in AGREE is a fraction of one, ranging from zero (least green) to one (highest green). The automatically generated pictogram has twelve pieces, each of which has a distinctive fundamental and their color ranges from deep green to deep red according to their agreement with the greenness. The AGREE tool gave our methods a score of 0.84 out of 1 proving the eco friendliness of the methods, Fig. 10c.

ESA and AGREE provided reliable numerical assessments that differed in their total scores whereas the total scores were out of 100 and 1 for each, respectively. AGREE has the merits over ESA with respect to automation and highlighting the weakest points in analytical techniques that need further improvements in terms of greenness. GAPI and AGREE provide fully descriptive three-colored pictograms. The main disadvantage of GAPI is complexity compared to NEMI and ESA. AGREE has the merits of simplicity and automation over GAPI. Based on the results (Fig. 10 and Table 4), the proposed methods adhered highly to the greenness characteristics of the four mentioned tools.

#### Sustainability assessment

The hexagon tool is a cutting-edge multicriteria technique that tries to completely evaluate analytical procedures and thus is considered a sustainability tool. The parameters under consideration are analytical performance, sustainability, eco-friendliness, and economic cost. They are inspected using the penalization principle. The overall evaluation is figured in a hexagonal pictogram through the following aspects:

1 Figures of merit (quality parameters) are divided into two categories: FM-1 figures of merit involving sample treatment, method features, and calibration procedure. Figures of merit 2 (FM-2) apply to the analytical method's quality control/verification and accuracy.

2 Toxicity and security represents the toxicity and risk of the chemical products used, as well as the analyst's safety.

3 Residues triangle, which evaluates the volume of waste generated and its treatment.

4 A carbon footprint triangle is used to assess the environmental impact.

5 Annual economic cost linked with the understudied analytical procedure: the higher the value, the poorer the cost/benefit ratio relation.

**Fig.11:**
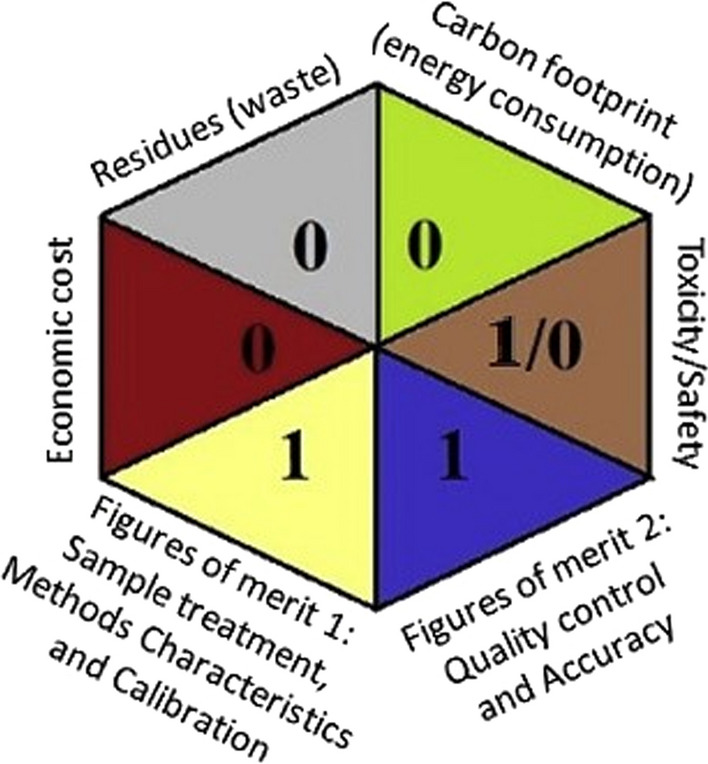
The Hexagon profile of the proposed spectrophotometric methods

Deviation from ideality is measured by penalty points (PP) for each of the evaluation factors, resulting in a final score ranging from 0 to 4. The assessment tables and overall evaluation table of each factor in relation to the PP ranges can be found in the original paper [59]. The factors under consideration are grouped in six equilateral triangles, each with a color associated with the criterion rather than the score. The technique with zeros and ones is of ultimate sustainability and environmentally friendliness. According to the hexagonal pictogram data, the proposed spectrophotometric approaches had 4 zeros and 3 ones, Fig. 11. The hexagon tool results confirmed the optimal performance of our procedure in various aspects such as toxicity, safety, produced waste, the use of energy, economic cost, and good performance in terms of analytical methodology validation.

#### Spider diagram for the assessment of the greenness index of the solvent used

In our novel proposed spectrophotometric methods, MeOH was evaluated utilizing the Greenness Index with spider diagram. This tool depends on data gathered from SDSs, which contain information regarding a solvent's characteristics and how they influence SHE throughout the process. Five assessment criterion subcategories were combined in a hierarchical spider diagram to generate a visual representation of the total sustainability degree for the chemicals used (Health Impact, Overall Characteristics, Odour, Fire Safety, and Stability). The results of these criteria ranged from − 5 to + 5. There are more spider charts with more details on each of the five subcategories mentioned above. Since many chemical reagents do not provide all of the necessary information for the five aforesaid subgroups on an SDS, this missing data obtained a 0 score in the calculation. The accessible data needed to calculate the Greenness Index values, as well as any missing data, are made available in the form of a table termed as the "Greenness Index Table." These references show the level of trustworthiness in the greenness assessment. [60–63]

**Fig. 12:**
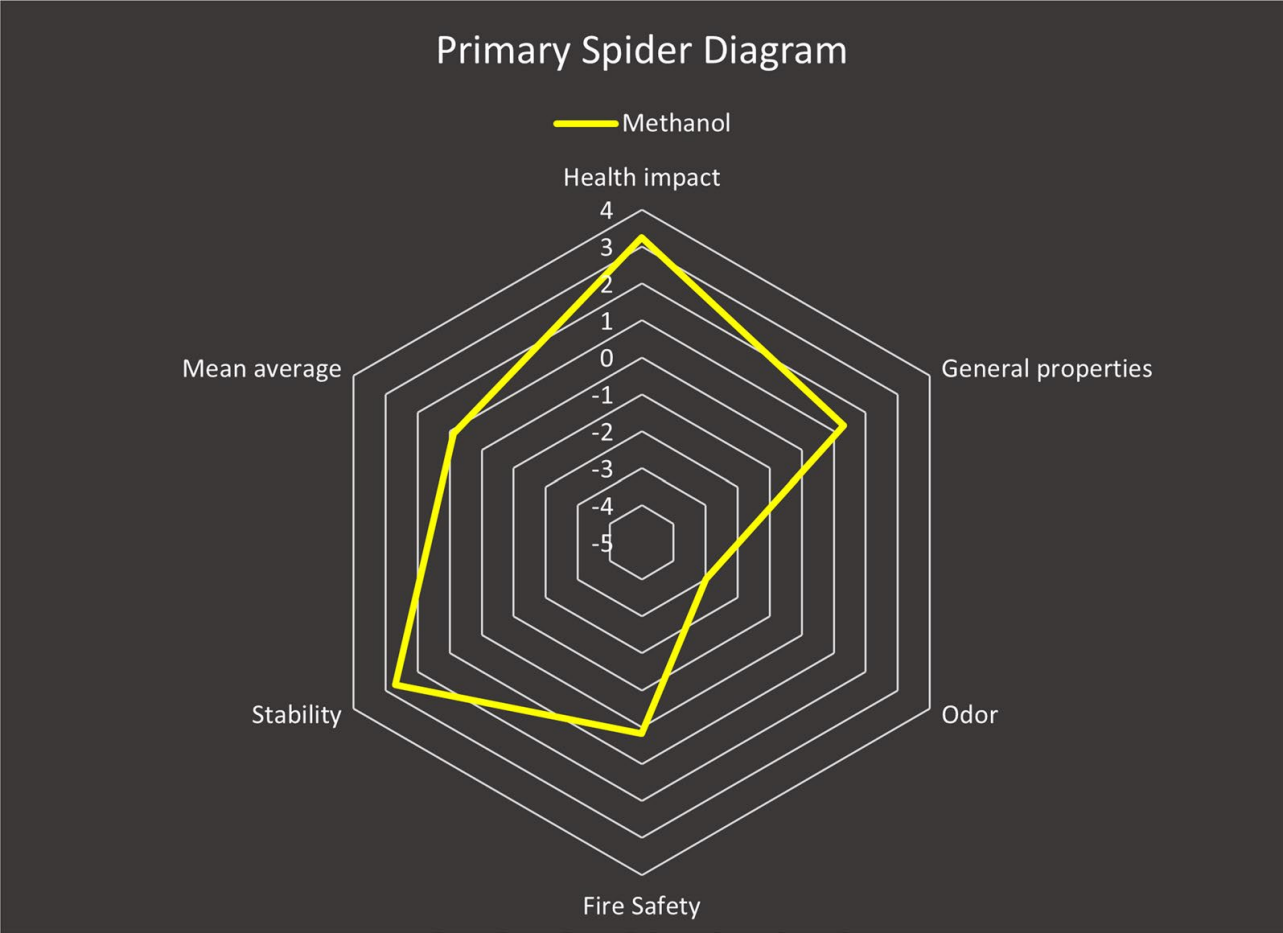
Primary spider diagram for MeOH

**Fig. 13:**
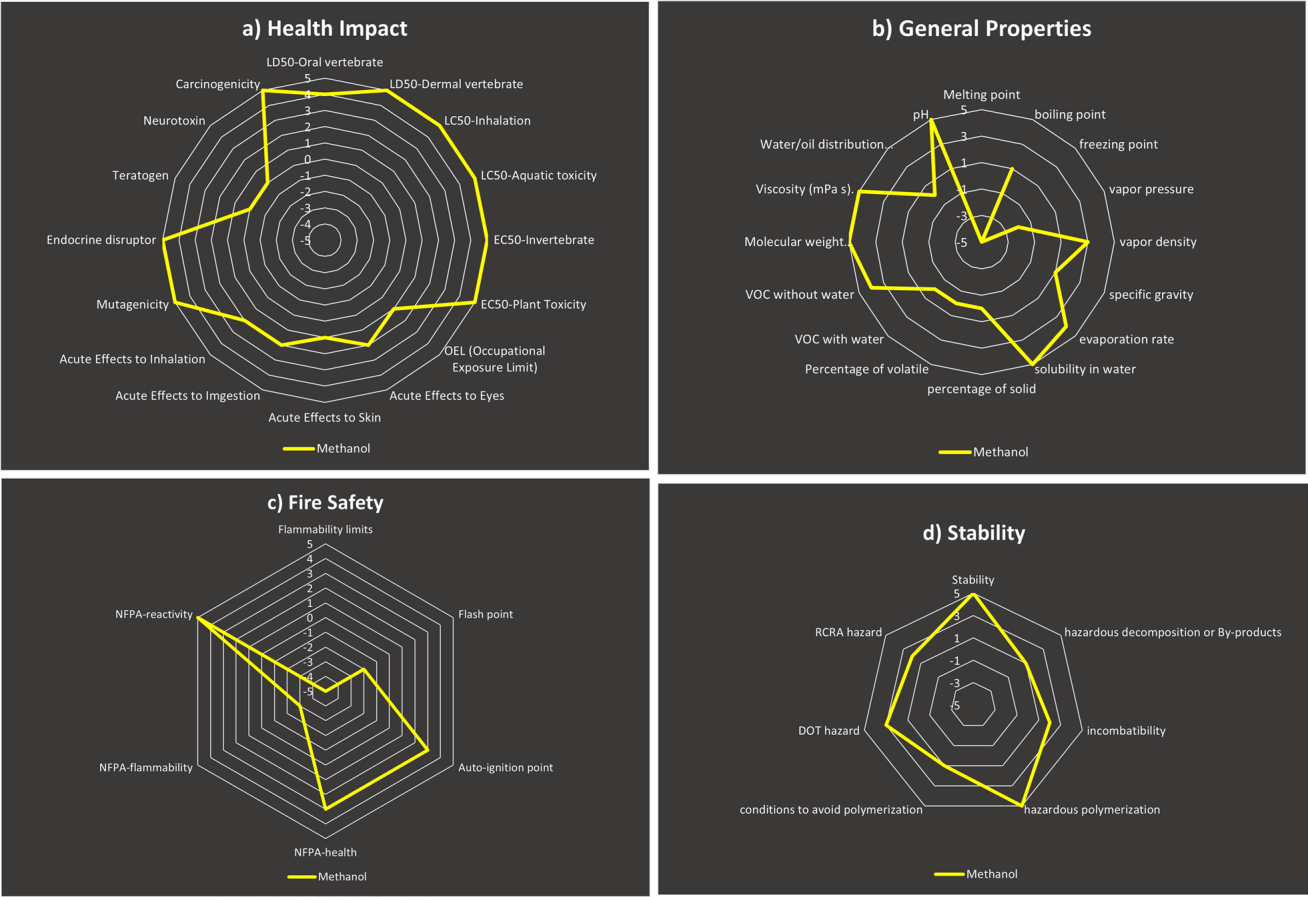
Secondary spider diagram for: **A**- Health impact, **B**- Stability, **C**- General properties, and **D**- Fire Safety for MeOH

This spider method allows visual reagent appraisal, making the comprehensive analysis of MeOH, used in our technique, simple and clear. The primary spider chart, Fig. 12, demonstrates that MeOH has a positive average score, 0.89, indicating their safety for the environment and human health. The supporting information for the other scores is displayed in the secondary spider charts in Fig. 13a–d. Table 5 Greenness Index displays the average scores and proportion of meaningful data for MeOH.

**Table 5:** Greenness Index Table for MeOH

	MeOH score	Available information
Health impact	3.25	100
General properties	1.31	87.5
Odor	− 3	100
Fire safety	0.17	100
Stability	2.71	100
Mean average	0.89	97.50

Although the Spider Greenness Index technique is difficult to adopt since it requires individual effort and entails studying numerous SDSs in order to obtain the most helpful information, it provides a visual depiction of the solvent greenness comparability by explaining the related sub-points with each greenness criterion using secondary spider charts.

### *Insilico* prediction of severity of DDIs

A hemeprotein called cytochrome P450 (CYP) is essential for the metabolism of medications. Given the potential severity of drug-drug interactions, advanced practitioners must have a thorough understanding of the CYP system. Drugs having CYP activity can change the metabolism of substances that are provided concurrently by acting as substrates, inducers, or inhibitors of a particular CYP enzymatic pathway. Medication toxicity can arise from pharmaceuticals that block a CYP enzymatic pathway because of possible increase in plasma concentration levels of CYP-substrates. Similarly, medications that trigger a CYP enzymatic pathway may lower the plasma concentration levels of medications metabolized by the same process, resulting in subtherapeutic drug levels or unsuccessful treatment outcomes. The CYP enzymes from family 1, 2, or 3 were implicated in the majority of hepatic metabolism. CYP3A4/5, CYP2C9, CYP2D6, and CYP2C19 were the most frequently engaged pathways, accounting for around 79% of the oxidation of these medications. Drug-drug interactions may result from concurrent medication therapy. As a result, knowledge of the CYP system is crucial for drugs’ safety [67].

**Fig. 14:**
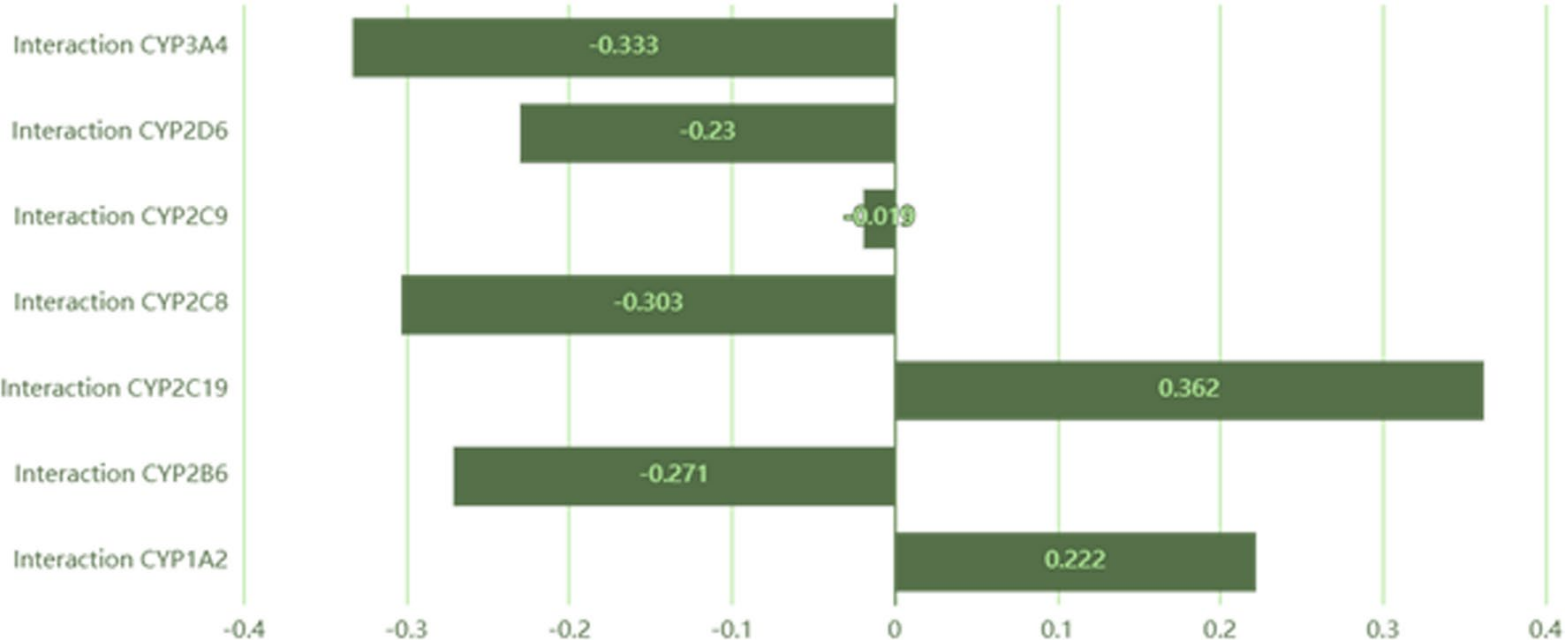
Diagram showing *IAP* values of DDIs between FVM and IVM at the level of the seven CYP450 enzymes

After adding the structures of the two compounds, FVM and IVM, in the Way2drug portal, DDIs mediated by CYP450 were evaluated. The *IAP* (Invariant Accuracy of Prediction) was calculated. The *IAP* is the difference between *Pa* (probability "to be active") and *Pi* (probability "to be inactive"). The results of *IAP* give an indication about the probability of DDI at each enzyme level of the CYP450 group. If the results are negative, this means that *Pi* > *Pa* and, with a high probability, there is no DDI at the cytochrome level of the considered drugs. On the other hand, if *IAP* is positive and below 0.7, there is weak evidence of DDI and could be neglected. But if *IAP* is positive and higher than 0.7, a significant probability of DDI does exist. Five CYP450 enzymes of FVM and IVM, CYP2B6, CYP2C8, CYP2C9, CYP2D6, and CYP3A4 had negative values of *IAP*: − 0.271, − 0.303, − 0.019, − 0.23, and − 0.333, indicating no interaction between FVM and IVM at these enzymes, Additional file 1: Fig. S5. The *IAP* values of CYP1A2 and CYP2C19 were 0.222 and 0.362, respectively, illustrating weak evidence of interaction as the *IAP* values were less than 0.7, Fig. 14.

The severity of DDIs was determined by the ORCA system and it was found in class 5 which indicates no interaction (Class 5). The severity of DDIs is dependent on a variety of drug properties, like: belonging to a therapeutic class (antibacterial agents, substances affecting the cardiovascular and nervous systems); the therapeutic windows of drugs (potent drugs are more frequently involved in DDIs); the severity of side effects that the drugs may exhibit; and the pathway of metabolism of the drugs (adverse effects of DDI increase if the substance is metabolized by a single DME that can be blocked by an inhibitor); and the pharmacokinetic profile of interacting drugs is critical (cytochrome P450 3A4 inhibitors can cause severe DDIs).

Hence, at the *insilico* level, the FVM and IVM could be safely co-administered without affecting their pharmacokinetics parameters. Our proposed *insilico* method can help prioritize drug discovery endeavors by guiding, but not replacing, either in vitro or in vivo experiments.

## Conclusion

The pressing need for SARS-CoV-2 therapeutics prompted researchers to employ drug repurposing strategy. This strategy contributes to the decrease in drug development risks, time, and costs. Moreover, combination therapies of repurposed drugs enhance viral inhibition by targeting multiple targets, varying modes of action, and countering complications induced by infection. One of these promising combinations is FVM and IVM. Therefore, simple analytical methods for FVM and IVM determination in their mixtures without any prior separation is needed for quality control purposes. In this respect, four green UV-methods were used: the dual-wavelength method (DWM), the Fourier function convolution of absorption spectra (FFAS), the Fourier function convolution of derivative spectra of absorption curves (FFDS), and the Fourier function convolution of ratio spectra of absorption curves (FFRS). For the analysis of a poly pill containing both drugs in an attempt of the management of COVID, the described methodologies could be successfully applied. Due to the use of less harmful and ecologically friendly solvents, the new spectrophotometric procedures could be effective substitutes for more difficult approaches. This was proved using different greenness and sustainability tools including NEMI, Eco-scale, GAPI, AGREE, Hexagon and spider diagram. And because of our constant pursuit of green analysis besides simplicity, we used *insilico* prediction technique for DDIs to ensure safe use of FVM and IVM with no pharmacokinetic interaction.

### Supplementary Information

**Additional file 1: Figure S1. **First derivative spectra of different concentrations of FVM (5, 10, 20 μg/mL) and IVM (2.5, 10, 15 μg/mL). **Figure S2. **Second derivative spectra of different concentrations of FVM (5, 10, 20 μg/mL) and IVM (2.5, 10, 15 μg/mL). **Figure S3. **Derivative ratio spectra (I) of different concentrations of FVM (5, 10, 20 μg/mL) using 10 μg/mL IVM as divisor. **Figure S4. **Derivative ratio spectra (II) of different concentrations of IVM (5, 10, 20 μg/mL) using 10 μg/mL FVM as divisor. **Table S1.** Summary of assay results and one–way analysis of variance (ANOVA) for the determination of FVM and IVM by FFAS, FFDS, FFRS, and DWZ.

## Data Availability

The datasets used and/or analysed during the current study available from the corresponding author on reasonable request.

## Abbreviations

FVM: Fluvoxamine
IVM: Ivermectin
LOD: Limit of detection
LOQ: Limit of quantification
GAPI: Green Analytical Procedure Index
AGREE: Analytical Greenness metric
DDIs: Drug-drug interactions
FFAS: Fourier functions convolution of absorption spectra
FFDS: Fourier functions convolution of derivative spectra of absorption curves
FFRS: Fourier function convolution of ratio spectra of absorption curves
DWM: Dual-wavelength method
NEMI: National Environmental Method Index

## References

[1] Poduri R, Joshi G, Jagadeesh G (2020). Drugs targeting various stages of the SARS-CoV-2 life cycle: exploring promising drugs for the treatment of Covid-19. Cell Signal.

[2] Huang C (2020). Clinical features of patients infected with 2019 novel coronavirus in Wuhan. China The Lancet.

[3] Pan L (2020). Clinical characteristics of COVID-19 patients with digestive symptoms in Hubei, China: a descriptive, cross-sectional Multicenter study. Am J Gastroenterol.

[4] Edwards K, Hussain I (2021). Two cases of severe autoimmune thyrotoxicosis following SARS-CoV-2 infection. J Investig Med High Impact Case Rep.

[5] Baig AM, Sanders EC (2020). Potential neuroinvasive pathways of SARS-CoV-2: Deciphering the spectrum of neurological deficit seen in coronavirus disease-2019 (COVID-19). J Med Virol.

[6] Du Y (2020). Clinical features of 85 fatal cases of COVID-19 from Wuhan. A retrospective observational study. Am J Respir Crit Care Med.

[7] Faqihi F (2020). Therapeutic plasma exchange in adult critically ill patients with life-threatening SARS-CoV-2 disease: a pilot study. J Crit Care.

[8] Alharthy A (2020). Lung injury in COVID-19-An emerging hypothesis. ACS Chem Neurosci.

[9] Grasselli G (2020). Baseline characteristics and outcomes of 1591 patients infected with SARS-CoV-2 admitted to ICUs of the Lombardy Region. Italy Jama.

[10] Jiang F (2020). Review of the clinical characteristics of coronavirus disease 2019 (COVID-19). J Gen Intern Med.

[11] Waqar W (2021). SARS-CoV-2 associated pathogenesis, immune dysfunction and involvement of host factors: a comprehensive review. Eur Rev Med Pharmacol Sci.

[12] Ruan Q (2020). Clinical predictors of mortality due to COVID-19 based on an analysis of data of 150 patients from Wuhan China. Intensive Care Med.

[13] Torres I (2020). COVID-19 vaccination: returning to WHO's Health For All. Lancet Glob Health.

[14] Rayner CR (2020). Accelerating clinical evaluation of repurposed combination therapies for COVID-19. Am J Trop Med Hyg.

[15] Govender K, Chuturgoon A (2022). An overview of repurposed drugs for potential COVID-19 treatment. Antibiotics.

[16] Omi T (2014). Fluvoxamine alleviates ER stress via induction of Sigma-1 receptor. Cell Death Dis.

[17] Sukhatme VP (2021). Fluvoxamine: a review of its mechanism of action and its role in COVID-19. Front Pharmacol.

[18] Hashimoto Y, Suzuki T, Hashimoto K (2022). Mechanisms of action of fluvoxamine for COVID-19: a historical review. Mol Psychiatry.

[19] Tynan RJ (2012). A comparative examination of the anti-inflammatory effects of SSRI and SNRI antidepressants on LPS stimulated microglia. Brain Behav Immun.

[20] Wang L (2019). Effects of SSRIs on peripheral inflammatory markers in patients with major depressive disorder: a systematic review and meta-analysis. Brain Behav Immun.

[21] Reis G (2022). Effect of early treatment with fluvoxamine on risk of emergency care and hospitalisation among patients with COVID-19: the TOGETHER randomised, platform clinical trial. Lancet Glob Health.

[22] Lenze EJ (2020). Fluvoxamine vs placebo and clinical deterioration in outpatients with symptomatic COVID-19: a randomized clinical trial. JAMA.

[23] Hoertel N (2021). Association between antidepressant use and reduced risk of intubation or death in hospitalized patients with COVID-19: results from an observational study. Mol Psychiatry.

[24] Formiga FR (2021). Ivermectin: an award-winning drug with expected antiviral activity against COVID-19. J Control Release.

[25] Biber A (2022). The effect of ivermectin on the viral load and culture viability in early treatment of nonhospitalized patients with mild COVID-19—a double-blind, randomized placebo-controlled trial. Int J Infect Dis.

[26] Yang SNY (2020). The broad spectrum antiviral ivermectin targets the host nuclear transport importin α/β1 heterodimer. Antiviral Res.

[27] Caly L (2020). The FDA-approved drug ivermectin inhibits the replication of SARS-CoV-2 in vitro. Antiviral Res.

[28] Rizzo E (2020). Ivermectin, antiviral properties and COVID-19: a possible new mechanism of action. Naunyn Schmiedebergs Arch Pharmacol.

[29] Sandler ZJ (2020). Novel Ionophores active against La crosse Virus Identified through rapid antiviral screening. Antimicrob Agents Chemother.

[30] Ebbelaar CCF, Venema AW, Van Dijk MR (2018). Topical ivermectin in the treatment of papulopustular rosacea: a systematic review of evidence and clinical guideline recommendations. Dermatol Ther.

[31] Chaccour C (2020). The SARS-CoV-2 ivermectin navarra-ISGlobal trial (SAINT) to evaluate the potential of ivermectin to reduce COVID-19 transmission in low risk, non-severe COVID-19 patients in the first 48 hours after symptoms onset: a structured summary of a study protocol for a randomized control pilot trial. Trials.

[32] Bocci G (2020). Virtual and in vitro antiviral screening revive therapeutic drugs for COVID-19. ACS Pharmacol Transl Sci.

[33] Luo Q, Zheng Y, Zhang J (2022). Combination therapies against COVID-19. Front Biosci.

[34] Clark, C. *Treatment of covid-19 with ivermectin + fluvoxamine combination*. 2021. https://medicalupdateonline.com/2021/06/treatment-of-covid-19-with-ivermectin-fluvoxamine-combination/. Accessed 18 Oct 2023.

[35] Dmitriev AV (2021). In silico prediction of drug-drug interactions mediated by cytochrome P450 isoforms. Pharmaceutics.

[36] Dmitriev A (2019). Prediction of severity of drug-drug interactions caused by enzyme inhibition and activation. Molecules.

[37] Dmitriev AV (2019). Drug-drug interaction prediction using PASS. SAR QSAR Environ Res.

[38] Devaka N, Rao V (2019). Chromatographic quantification of ivermectin and pranziquantel in the tablets using stability indicating RP-HPLC method. Pharm Sci.

[39] Taspinar N (2023). Comparation with spectrophotometric and liquid chromatographic methods of pharmaceutical forms of Ivermectin. Med Records.

[40] Varghese SJ, Vasanthi P, Ravi TK (2011). Simultaneous densitometric determination of Ivermectin and albendazole by high-performance thin-layer chromatography. JPC J Planar Chromatogr Modern TLC.

[41] Madhan S, Kavitha J, Ks L (2019). Multivariate calibration technique for the spectrophotometric quantification of ivermectin in pharmaceutical formulation. Asian J Pharm Clin Res.

[42] Hemke AT, Gupta KR (2015). Force degradation study and rp-hplc method development for estimation of fluvoxamine maleate in tablet. Int J Pharm Pharm Sci.

[43] Ali I (2013). Separation and Identification of antidepressant drugs in human plasma by solid-phase extraction-thin-layer chromatography. JPC J Planar Chromatogr Modern TLC.

[44] Elmalı F (2000). Polarographic determination of fluvoxamine maleate in tablets. Turk J Chem.

[45] Hashem HM (2020). Cost-effective potentiometric platforms modified with multi-walled carbon nanotubes (MWCNTs) and based on imprinted receptors for fluvoxamine assessment. Polymers.

[46] Darwish IA (2009). Spectrofluorimetric determination of fluvoxamine in dosage forms and plasma via derivatization with 4-Chloro-7-Nitrobenzo-2-Oxa-1,3-Diazole. J Fluoresc.

[47] Abu-Hassan AA, Omar MA, Derayea SM (2020). New approach for stability study and determination of fluvoxamine in raw materials and pharmaceuticals through condensation with 2,2-dihydroxyindane-1,3-dione. Luminescence.

[48] Darwish IA (2009). Spectrophotometric study for the reaction between fluvoxamine and 1,2-naphthoquinone-4-sulphonate: kinetic, mechanism and use for determination of fluvoxamine in its dosage forms. Spectrochim Acta A Mol Biomol Spectrosc.

[49] Annapurna V (2010). Spectrophotometric methods for the assay of fluvoxamine using chromogenic reagents. J Chem.

[50] Sayed RA (2021). Non-extractive spectrophotometric determination of memantine HCl, clomipramine HCl and fluvoxamine maleate in pure form and in pharmaceutical products by ion-pair complex formation with rose bengal. Ann Pharm Fr.

[51] El-Awady MI (2023). Multicomponent spectrophotometric determination of a ternary mixture of widely-prescribed cardiovascular drugs by four different methods. Spectrochim Acta Part A Mol Biomol Spectrosc.

[52] El-Kimary EI (2020). Fourier convolution versus derivative spectrophotometry: Application to the analysis of two binary mixtures containing tamsulosin hydrochloride as a minor component. Ann Pharm Fr.

[53] Youssef RM, Maher HM (2008). A new hybrid double divisor ratio spectra method for the analysis of ternary mixtures. Spectrochim Acta A Mol Biomol Spectrosc.

[54] Fawzy MG (2023). Green-assisted spectrophotometric techniques utilizing mathematical and ratio spectra manipulations to resolve severely overlapped spectra of a cardiovascular pharmaceutical mixture. Spectrochim Acta Part A Mol Biomol Spectrosc.

[55] Keith L (2005). An introduction to the national environmental methods index. Environ Sci Technol.

[56] Gałuszka A (2012). Analytical eco-scale for assessing the greenness of analytical procedures. TrAC, Trends Anal Chem.

[57] Płotka-Wasylka J (2018). A new tool for the evaluation of the analytical procedure: green analytical procedure index. Talanta.

[58] Pena-Pereira F, Wojnowski W, Tobiszewski M (2020). AGREE—analytical GREEnness metric approach and software. Anal Chem.

[59] Ballester-Caudet A (2019). A new tool for evaluating and/or selecting analytical methods: summarizing the information in a hexagon. TrAC, Trends Anal Chem.

[60] Abou-Taleb NH (2021). Spider diagram and Analytical GREEnness metric approach for assessing the greenness of quantitative 1H-NMR determination of lamotrigine: Taguchi method based optimization. Chemom Intell Lab Syst.

[61] Shen Y (2016). Development of greenness index as an evaluation tool to assess reagents: evaluation based on SDS (Safety Data Sheet) information. Miner Eng.

[62] Lotfy HM, Obaydo RH, Nessim CK (2023). Spider chart and whiteness assessment of synergistic spectrophotometric strategy for quantification of triple combination recommended in seasonal influenza—Detection of spurious drug. Sustain Chem Pharm.

[63] Kayali Z, Obaydo RH, Alhaj Sakur A (2023). Spider diagram and sustainability evaluation of UV-methods strategy for quantification of aspirin and sildenafil citrate in the presence of salicylic acid in their bulk and formulation. Heliyon.

[64] *DDI-Pred: Web-Service for Drug-Drug Interaction Prediction*. http://way2drug.com/ddi/. Accessed 22 July 2023.

[65] International Conference on Harmonisation (ICH). *ICH, Validation of Analytical Procedures: Text and Methodology, Q2(R1)*. ICH; 2005.

[66] Miller JN, Miller JC (2005). Statistics and Chemometrics for Analytical Chemistry.

[67] McDonnell AM, Dang CH (2013). Basic review of the cytochrome p450 system. J Adv Pract Oncol.

