# Supplementary material for: Utility of sustainable ratio derivative spectrophotometry for the concurrent assay of synergistic repurposed drugs for COVID-19 infections; Insilico pharmacokinetics proof
Source: BMC Chem. 2024 Mar 7;18(1):50. doi: 10.1186/s13065-024-01147-w (PMC10921645; doi:10.1186/s13065-024-01147-w)
Supplement: Supplementary file 1 — Additional file 1: Figure S1. First derivative spectra of different concentrations of FVM (5, 10, 20 μg/mL) and IVM (2.5, 10, 15 μg/mL). Figure S2. Second derivative spectra of different concentrations of FVM (5, 10, 20 μg/mL) and IVM (2.5, 10, 15 μg/mL). Figure S3. Derivative ratio spectra (I) of different concentrations of FVM (5, 10, 20 μg/mL) using 10 μg/mL IVM as divisor. Figure S4. Derivative ratio spectra (II) of different concentrations of IVM (5, 10, 20 μg/mL) using 10 μg/mL FVM as divisor. Table S1. Summary of assay results and one–way analysis of variance (ANOVA) for the determination of FVM and IVM by FFAS, FFDS, FFRS, and DWZ. [file 13065_2024_1147_MOESM1_ESM.docx]

Supplementary figures:

**Supp Fig 1.** First derivative spectra of different concentrations of FVM (5, 10, 20 μg/mL) and IVM (2.5, 10, 15 μg/mL).

**Supp Fig 2.** Second derivative spectra of different concentrations of FVM (5, 10, 20 μg/mL) and IVM (2.5, 10, 15 μg/mL)

**Supp Fig. 3. Derivative** ratio spectra (I) of different concentrations of FVM (5, 10, 20 μg/mL) using 10 μg/mL IVM as divisor.

**Supp Fig. 4. Derivative** ratio spectra (II) of different concentrations of IVM (5, 10, 20 μg/mL) using 10 μg/mL FVM as divisor.

Table S1: Summary of assay results and one–way analysis of variance (ANOVA) for the determination of FVM and IVM by FFAS, FFDS, FFRS, and DWZ.

|  | **Source of Variation** | **SS** | **Df** | **MS** | **F** | **P-value** | **F crit** |  |
| --- | --- | --- | --- | --- | --- | --- | --- | --- |
|  |  |  |  |  |  |  |  |  |
| **FVM** | Between Groups | 0.82 | 2 | 0.41 | **3.55** | 0.10 | **5.14** |  |
|  | Within Groups | 0.69 | 6 | 0.11 |  |  |  |  |
|  | Total | 1.51 | 8 |  |  |  |  |  |
|  | Between Groups | 0.75 | 2 | 0.38 | **0.36** | 0.71 | **5.14** |  |
| **IVM** | Within Groups | 6.21 | 6 | 1.04 |  |  |  |  |
|  | Total | 6.96 | 8 |  |  |  |  |  |
